# When time matters: generalization gradients in delay and trace conditioning procedures

**DOI:** 10.3758/s13420-026-00708-4

**Published:** 2026-02-02

**Authors:** José A. Alcalá, Jessica C. Lee, Gonzalo P. Urcelay

**Affiliations:** 1 Facultad de Ciencias de la Salud, Universidad Rey Juan Carlos, Madrid, Spain; 2 Department of Psychology, University of Sydney, Sydney, NSW Australia; 3 Department of Psychology, University of New South Wales, Sydney, NSW Australia; 4 School of Psychology, University of Nottingham, University Park, Nottingham, NG7 2RD United Kingdom

**Keywords:** Delay, Trace, Generalization gradients, Predictive learning

## Abstract

Two online experiments investigated whether the weakening of cue-outcome temporal contiguity impacts generalization gradients. Participants experienced strong temporal contiguity (the offset of a cue coincides with the onset an outcome) or weak temporal contiguity (there is a temporal gap between the offset of a cue and the onset of an outcome) in a continuous predictive learning task. Initial training involved a double negative discrimination, in which there was one reinforced cue (aqua-color rectangle) and two non-reinforced cues (one greener and one bluer than the reinforced cue), and participants had to make a response whenever they predicted the outcome. After training, in the generalization test phase, outcome expectancy in the presence of several stimuli along the full green-blue dimension was tested. Outcome expectancy was assessed with discrete ratings in Experiment 1 (*n* = 252) and a behavioral dependent measure (button presses, similar to the training phase) in Experiment 2 (*n* = 250). Regardless of the type of measure, when participants experienced weak cue-outcome temporal contiguity they showed a broader generalization response, although differences were more prominent in the green side of the color dimension. Furthermore, no significant differences emerged between the two groups experiencing strong temporal contiguity but differing in cue duration (controlling stimulus onset asynchrony, SOA), suggesting that cue-outcome temporal contiguity plays a more critical role than cue duration in shaping generalization. Overall, this result advances our understanding of the role of temporal contiguity at a basic level, while opening the possibility to study it as a critical factor in more applied settings.

## Introduction

The temporal relationship between events is a critical determinant of learning. A cue that is immediately followed by an outcome is easily identified as its predictor. However, as the temporal interval between the cue and the outcome increases, behavioral control exerted by the cue decreases (e.g., impaired outcome expectancy). In daily life, people experience instances of both strong and weak temporal contiguity. For example, a snack retrieved from a vending machine after payment exemplifies an example of strong temporal contiguity between the action of paying and the delivery of the snack, whereas receiving a food delivery at home many hours after ordering it online represents an instance of weak temporal contiguity between an action and outcome receipt.

The impact of variations in temporal contiguity on learning has historically been a relevant question for basic learning theorists (see Boakes & Costa, 2014, for a review). When studying contiguity, researchers usually compare a *delay conditioning* procedure (in which the offset of the cue coincides with the onset of the outcome) with a *trace conditioning* procedure (in which there is a temporal gap between the offset of the cue and the onset of the outcome). Learning usually proceeds faster with delay conditioning than with trace conditioning. For instance, rats demonstrate more fear anticipating a mild foot shock when it immediately followed a positive conditioned stimulus (CS+) compared to when there is a temporal gap between CS+ and the unconditioned stimulus (US) (Kamin, 1961). Therefore, weakening temporal contiguity impairs learning.

In a scenario involving discrimination learning (CS+ vs. CS˗), the general pattern of results is similar: weakening of temporal contiguity impairs acquisition of the discrimination. For instance, Honey and Hall (1992) trained rats to discriminate between two auditory stimuli. One group of animals was trained with delay conditioning, whereas the other group experienced a 30-s trace between the CS+ termination and presentation of a mild foot shock. Animals in group Delay showed good discrimination between stimuli, that is, they showed fear toward the CS+, but not toward the CS˗. On the contrary, animals in group Trace showed similar levels of fear toward both cues, in other words little discrimination between stimuli (also see Ellison, 1964; Pavlov, 1927). However, this effect is not universal, for example in humans the discrimination seems to depend on other factors such as participant awareness (Lovibond et al., 2011), task demands (Carter et al., 2003), trace duration (Reynolds, 1945), expectations (Buehner & May, 2004), and events intervening during the trace interval (Costa & Boakes, 2011). Moreover, the design and type of measure employed are critical to observe the deleterious effect of trace conditioning, given that not all measures of learning are sensitive to standard trace-conditioning procedures (Wehrli et al., 2022). Therefore, in the case of human learning, variations in temporal contiguity do not always result in differences in responding.

Beyond the effects on the rate of acquisition and associative strength, variations in temporal contiguity can also alter behavioral control exerted by specific cues trained as part of a compound, and the cue-competition literature provides substantial evidence of this. Overshadowing is a typical case in which two cues (AX) are presented together and paired with the outcome. Typically, the behavioral control yielded by the target cue (X) is attenuated by the presence of A (usually more salient than the target), compared to a situation in which only a control cue (Y) is associated with the outcome. Therefore, the presence of A overshadows behavioral control by X. Notably, the overshadowing effect only occurs when there is strong temporal contiguity between cues and outcomes (e.g., Urcelay & Miller, 2009), and is attenuated when contiguity is weak. In the latter case, target and control cues yield similar behavioral control (Alcalá, Miller, et al., 2023, Alcalá, Ogallar, et al., 2023; Herra et al., (Herrera, et al., 2022) Furthermore, under certain conditions overshadowing training can result in facilitation (i.e., potentiation), where a target event trained in combination with another event exerts greater control than when trained in isolation (Alcalá, Kirkden, et al., 2023; Cunha et al., 2015; Urcelay & Miller, 2009; Vila et al., 2024). Hence, the interaction between events (overshadowing or potentiation) seems to depend on temporal contiguity.

A classic account of overshadowing is the configural theory of Pearce (1987), which posits that the observation of overshadowing results from generalization decrement from compound training (AX) to elemental testing (X). Given that AX and X are dissimilar, responding to X at test presumably results from this dissimilarity. In other words, because X is different from AX, the response of the organism is attenuated. However, this account is silent about instances in which the interaction between cues is facilitative instead of competitive. This raises the question of why – in the observations of potentiation – the weakening temporal contiguity results in an increase in responding to X during test.

Herrera and colleagues (2022; also see Urcelay, 2017) recently suggested a potential explanation, which posits that the generalization decrement wanes (i.e., generalization response increases) as temporal contiguity weakens, possibly due to the “forgetting” of stimulus attributes over time (Riccio et al., 1994). This modification of Pearce’s model suggests that temporal contiguity plays a vital role in generalization and discrimination. That is, weaker temporal contiguity increases generalization from compound AX to isolated X, attenuating the overshadowing effect and sometimes resulting in potentiation of X by AX training with the use of trace procedures (Alcalá, Kirkden, et al., 2023; Batsell, et al., 2012; Urcelay & Miller, 2009). The broader generalization with the use of trace procedures might also explain why trace-conditioned animals displayed less discrimination between CS+ and CS˗, as observed in Honey and Hall’s study (1992). Indeed, these results can be interpreted as an instance of broader generalization, because with a trace procedure they not only observed attenuated responding to the CS+, but also observed increased responding to the CS˗ (relative to the delay-conditioned animals). However, in these cases (also see Elison, 1964), the generalization of responding was restricted to the trained stimuli, as they did not test whether manipulation of contiguity had an effect on novel stimuli, a key aspect of generalization.

Thus, previous empirical work suggests that weakening contiguity broadens generalization (also see Pavlov, 1927), and the recent theorizing by Herrera and colleagues (2022, 2025) also poses that this is the case. From a general perspective, generalization can be understood as the process by which organisms use previous experience to guide future responses in the presence of novel stimuli. Seminal research by Guttman and Kalish (1956) established that conditioned responding generalizes in a graded fashion along a continuous stimulus dimension, producing a smooth, bell-shaped gradient. In a discriminated operant preparation, pigeons were trained to peck at a visual cue with a particular wavelength to obtain food as a reinforcer, which resulted in the pigeons pecking the key with the color. In subsequent tests, they systematically varied the wavelength of the visual cue and documented the generalization gradients (strongest conditioned response typically occurred at the trained wavelength, with progressively weaker responses as cues deviated from the trained CS+ value). This pattern has been consistently observed across species and learning modalities (see Ghirlanda & Enquist, 2003; Shepard, 1987).

The shape of the generalization gradient tends to reflect the behavioral control yielded by a particular dimension of the stimuli. Such dimensions can be related with physical properties of the stimuli (e.g., color; Lovibond et al., 2020), affective features (Alcalá et al., 2024), or social features (Dunsmoor et al., 2009), among others. Depending on specific factors, the gradient can result in a steeper or broader shape according to variations in a particular dimension. For instance, in a single cue learning, a steeper gradient indicates that non-trained stimuli varying along that dimension elicit little behavioral control, and consequently a very sharp discrimination. On the contrary, broader gradients reveal less stimulus control by the varying dimension, to the extent that a flat gradient reflects no control by the varying dimension (Hovland, 1937).

Given the large tradition of basic research exploring the role of temporal contiguity manipulations and the fact that generalization is increased in participants with anxiety disorders (Beckers et al., 2023; Lissek et al., 2014), it is surprising that there is a dearth of studies exploring how temporal contiguity may affect generalization gradients. Indeed, to the best of our knowledge, we are not aware of any study directly exploring generalization gradients as a function of temporal contiguity in human participants. Therefore, the current study is concerned with this question, in that we explored whether weakening of temporal contiguity broadens the shape of generalization gradients. Thus, the goals of this study were twofold. Firstly, building on the previous literature, we aimed to assess whether training with trace conditioning broadens generalization gradients. As noted above, differences between delay and trace conditioning are not always observed in human participants. However, even in a scenario in which differences between delay and trace conditioning are not observed during acquisition, it could be possible that there will be differences during a generalization test. For instance, in a predictive learning experiment exploring generalization responses, Lee and colleagues (2018) reported that participants successfully discriminated between a CS+ and CS˗ at the end of training. However, during a generalization test, some participants engaged in a linear rule based on the color dimension of the stimuli (e.g., the greener the color the more likely the outcome), while others responded to generalization stimuli based on their physical resemblance to the CS+. That is, after similar levels of discrimination, there were differences in how participants responded to novel stimuli, resulting in different gradients.

Secondly, a popular account for the deleterious effect of trace conditioning is “trace decay theory,” in which there is a weakening of the memory representation of the CS during the trace. Such loss of stimulus attributes may lead to a decrement in the strength of the response (see Riccio et al., 1994). To this extent, we wanted to evaluate to what extent potential differences between delay and trace conditioning result from differences in perceptual discrimination of the stimuli during training (also see Lovibond et al., 2020).

## Experiment 1

Similar to studies in animals, we used a continuous-learning task (e.g., Honey & Hall, 1992). Participants made predictive responses upon presentation of three stimuli varying in color. We used a double-negative discrimination in which one CS+ (located in the middle of a green-blue dimension; i.e., aqua-color stimulus) was predictive of an outcome, whereas two equidistant CS˗ (one greener and one bluer stimulus) at both sides of the dimension were not predictive of the outcome. This design allowed us to find a symmetric pattern of generalization on both sides of the continuum during the test. Additionally, this design prevented participants from adopting a linear rule “the more blue/green the stimulus, the higher the likelihood of the outcome.” For example, a rule such as “the greener the stimulus, the more likely the outcome” (cf. Lee et al., 2018, where only one CS˗ was used) is incompatible with the present design, because one of the CS˗ is greener than the CS+. Consequently, any linear rule along the hue dimension would necessarily assign incorrect predictions to stimuli beyond the CS+, where the second CS˗ is placed. Therefore, successful performance must rely on discriminating stimuli according to their perceptual similarity to the CS+. Such a similarity-based strategy should yield a peaked (Gaussian) generalization gradient, with maximal responding to the CS+ and progressively lower responding to stimuli on both sides of the continuum (see Ng et al., 2022).

**Fig. 1:**
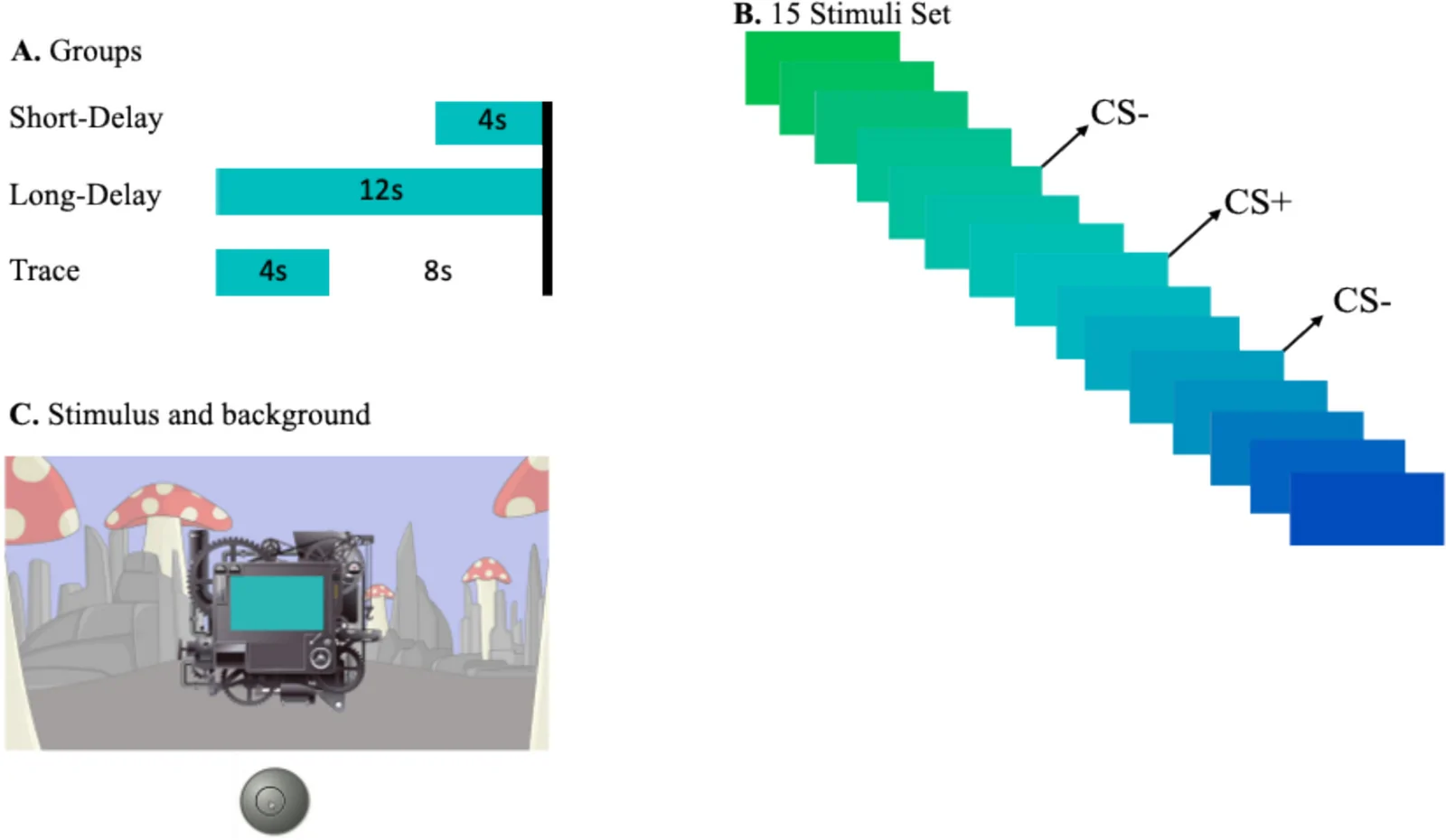
Experimental task, stimuli, and groups. (**A**) The groups in the experiments, with the duration of the cues. The black line represents the appearance of the outcome (i.e., lightning). (**B**) The stimuli used during expectancy and identification tests. (**C**) A snapshot of the learning task used in these experiments with the CS+ in the screen, the background and the button that participants clicked on to predict the outcome

Three groups of participants took part in the experiment with variations in the temporal relationship between CS+ and outcome (see Fig. 1A). In group Short-Delay, cues were presented for 4 s and the outcome was presented immediately after the offset of the CS+. In group Long-Delay, training was similar except that the cues lasted 12 s. In group Trace, the cue was displayed for 4 s (similar to group Short-Delay); however, there was a temporal gap of 8 s before the outcome appeared on the screen. We included two delay groups because each group controls for a specific issue. The stimulus-onset asynchrony (SOA) between cue and outcome differed between group Short-Delay and group Trace, and this might create differences in learning of each stimulus. To control for this issue, we included group Long-Delay (e.g., Barnet & Hunt, 2005). Although group Long-Delay had three times more exposure to the stimulus during training than group Trace, both groups had a similar number of presentations of the CS (12) and SOA between cue and outcome. Including both groups Delay can help to disentangle the nature of potential differences between groups.

In the generalization test, which was administered immediately after training, all groups were randomly exposed to 12 novel stimuli (intermixed with three previous trained stimuli: the CS+ and both CS˗ conditions), varying in the blue-green color dimension (see Fig. 1B). Based on the rationale described in the *Introduction*, we anticipated a broader generalization gradient (i.e., higher outcome expectancy across non-trained stimuli) in trace compared to delay conditioning.

Finally, we measured the participant’s ability to identify the stimulus in the first phase that predicted the outcome. This phase was aimed at understanding whether the expected broader generalization is accounted for by a general increase in the outcome expectancy or, contrarily, by the absence of discrimination (see Lovibond et al., 2020; also see Zaman et al., 2021).

### Method

#### Participants

Two hundred and fifty-two participants (146 female, 105 men, one non-binary) with an average age of 32.81 (*SD* = 9.83, range 18–64) years were recruited via Prolific in exchange for £2.5. The participants had no previous experience with the task. Participants were required to have completed more than 200 studies in Prolific with an approval rate > 98% and to be fluent in the English language. The study was approved by the Ethics Committee at the University of Nottingham.

#### Apparatus and materials

The task was programmed using the Gorilla Experiment Builder (Anwyl-Irvine et al., 2020). The task was inspired by and used similar materials to those used by Lovibond and colleagues (2020).

The stimuli were presented within an image of a machine, overlaid on an image of a fictitious city as the background (see Fig. 1C). Color rectangles of 200 × 100 pixels approximately were used as stimuli. The rectangles varied in their hue values between 0.4 and 0.6, keeping saturation and brightness at constant. Fifteen different stimuli were used in the experiment. The CS+ (S11) was in the middle of the continuum with a hue value of 0.5. The CS1˗ (S8; hue 0.47) and the CS2˗ (S14, hue 0.53) were either greener or bluer compared to the CS+. The number of each stimulus represents an increase of 0.01 in hue, from the greenest stimulus (S1, 0.40 hue) to the bluest (S21, 0.60 hue) (see Fig. 1B). In the absence of stimuli, the machine display showed a neutral gray (RGB: 120, 120, 120; HSL: 0, 0, 0.47).

A picture of a lightning bolt above the machine was used as the outcome. There was a round gray-button below the picture of the city that participants could press by clicking the mouse. During the training phase, the city background, the machine, and the button were always present. When participants pressed the button there was a small change in the brightness of the button, providing visual feedback for their responses.

#### Procedure

Participants were randomly allocated to groups Long-Delay, Short-Delay, or Trace. The order of phases was similar for all participants: Training > Expectancy Test > Identification Test. We did not counterbalance the order of the two tests because previous research using the same stimuli has shown extreme carry-over effects (Lovibond et al., 2020).

After signing the consent form and collection of demographic data, the following instructions were displayed:

- *[Screen1]. Imagine that you are working as an environmental scientist in the year 2137. Due to environmental contamination, there is a lower concentration of oxygen in the air. Fortunately, scientists have developed a new prototype of giant mushrooms that increase the levels of oxygen. These mushrooms make human life possible in many cities. You can see one city in the picture below.*
- *[Screen2]. Unfortunately, these mushrooms are very sensitive to lightning, and they will be damaged unless a security protocol is activated before lightning. Your mission in this experiment is to protect the mushrooms. Your colleagues have also created a machine that is able to predict when lightning is going to appear, so that the security team can activate a shield for the mushrooms. You can see the lightning and the machine below!*
- *However, this machine also displays a signal about other environmental parameters; hence, not all signals are informative about lightning. Sadly, your colleagues forgot to tell you what signal predicts the lightning! So, your task is to learn which signal predicts the lightning and to alert your colleagues about the lightning. You will see the same signal multiple times, so you will have several opportunities to try to find out which signal predicts the lightning.*
- *[Screen3]. There is a button below the machine that you should press every time you want to send an alert to the security team. The security team will use the number of presses as an indicator of whether or not to activate the shield for the mushrooms. In other words, the number of presses should reflect the strength of your belief about whether lightning is going to occur: the more you press the button, the more you believe lightning is coming. You will not see the shield being activated, but remember that your task is to alert your colleagues.*
- *[Screen4]. During the task, pay attention to the signal on the machine and when lightning occurs. When you think lightning will occur, alert the security team by pressing the button. Bear in mind that the security team will activate the protocol based on the number of alerts they receive, so try to alert them only when you think lightning is going to occur and not all the time!!*^1^ The safety of the city is in your hands!

Two attentional checks were included at the end of instructions. When participants failed one of the checks, they returned to the first screen and had to read the instructions again. This occurred until they passed both checks indicating that they had understood the instructions, otherwise they were not allowed to start the experiment.

##### Training phase

The training phase comprised 12 presentations of each stimulus (12 CS+, 12 CS1˗, 12 CS2˗). The CS+ (i.e., aqua-green stimulus, S11) was probabilistically associated with the outcome according to a programmed contingency of 0.75: nine of the 12 trials were followed by the outcome. The CS1˗ (a greener stimulus, S8) and the CS2˗ (a bluer stimulus, S14) were deterministically associated with the absence of the outcome. Trials were presented in three blocks of trials with four random presentations of each stimulus in each block, without any restrictions.

The stimuli appeared for a fixed amount of time. In the case of groups Short-Delay and Trace, stimuli lasted 4 s, while in group Long-Delay the stimuli were presented for 12 s. After the offset of the stimuli, the outcome (i.e., lightning) appeared immediately in both Delay groups, however, there was a temporal gap of 8 s in group Trace. During the trace, participants could continue pressing the alerting button. The outcome was symbolized by a yellow lightning symbol which appeared above the machine and lasted for 3 s. The inter-trial interval was 12 s for groups Long-Delay and Trace, and 20 s for group Short-Delay. In all cases the inter-trial interval (ITI) was variable (±  2 s). After finishing training, participants started the expectancy phase. 

##### Expectancy phase

Participants received the following instructions at the beginning of this phase.

*This is the second phase of the experiment. For the next few trials, you will be shown some more signals, but you will NOT be shown feedback about whether a lightning occurred. You should continue making predictions about whether you think a lightning will occur. However, in this phase, you will be making your prediction on a scale ranging from “Definitely NO LIGHTNING” to “Definitely LIGHTNING.” Use your mouse to drag the slider along the scale to make your rating.*

In this expectancy test participants received 15 stimuli in a random order without feedback about the outcome (e.g., Lee et al., 2018; Lovibond et al., 2020). Of note, this instruction avoids the potential effect of extinction while testing several stimuli without outcome (Lee et al., 2022). On each trial, a stimulus appeared with the question, “*The signal above appears on the machine. What is the likelihood of this stimulus leading to lightning?*” The horizontal scale ranged from 0 (“*Definitely NO LIGHTNING*”) to 100 (“*Definitely LIGHTNING*”). Initially, the pointer of the slider appeared on the middle, and participants dragged the pointer to the left or right. They needed to press the button “Next” to confirm the rating. Participants were not allowed to continue to the next question unless they moved the slider and pressed “Next.” There was no time limit to respond, and the ITI was 2 s. To avoid generalization decrement resulting from removing contextual cues, the stimulus was embedded with the machine and the city background, keeping the same display and size of the training phase. The slider was presented below the image of the city.

##### Identification phase

After the expectancy test, all participants received an identification test with the following instructions:

*This is the third phase of the experiment. For the next few trials, you will be shown some more signals. In the FIRST phase of the experiment, you saw only ONE SIGNAL that led to lightning. We will now ask you to IDENTIFY whether each signal is the SAME as or DIFFERENT from the one that led to shock in PHASE ONE.*

*Press “S” if you think the signal is the SAME as the signal that led to lightning in Phase ONE, or press “D” if you think the signal is DIFFERENT to the signal that led to lightning in Phase ONE*

This phase was similar to the expectancy test, except for the question “*Is the signal above the SAME (press S) as the one that led to LIGHTNING in phase 1 or is it DIFFERENT (press D)?*” Participants advanced to the next question after pressing S or D.

After each identification response, we asked participants to evaluate their confidence in their response using a scale from 1 to 9, in which 1 = “Not sure” and 9 = “Completely sure.” These data are reported in the Online Supplementary Material.

After all cues were tested in the identification test, we asked participants about self-reported attention check during the task with the following question:

*Well done! The experiment is over. Just one last question. Did you give your full attention to the experimental task (as opposed to sometimes doing other things like using your smartphone) while stimuli were being presented? Please answer honestly, this question has no impact on your payment. There are two options below, “Yes” and “No.”*

#### Data exclusion

We used several attentional and learning exclusion criteria to ensure data quality. In the case of attentional checks, participants were removed from the analyses in the following scenarios: (a) they reported being color-blind, (b) they declared not paying their full attention with the self-reported attention check. All participants reported not being color-blind. Seventeen participants declared not giving their full attention and were removed from the analyses. 235 participants passed the attentional checks.

In the case of learning criteria, participants were removed if they failed to acquire the relationship between stimuli and outcome during the training phase. Of note, excluding poor learners is standard practice in generalization experiments because it confounds interpretation of broader gradients (see Alcalá et al., 2024; Lee et al., 2018). Hence, to meet the learning criterion, participants needed to have a higher rate of responses to the CS+ than to the average of both CS˗ stimuli in the last block of training. Sixty-nine participants failed to pass the learning criteria and were not included in the analyses. The final sample consisted of 61 participants in group Long-Delay, 61 in group Short-Delay, and 52 in group Trace.

#### Data analyses

During training we recorded the average rate of pressing predicting the shock in each of the trained CSs, and calculated a discrimination index. We computed the discrimination index by dividing the proportion of responses to the CS+ in each block of training with the average of responding to both non-reinforced stimuli CS˗ plus the response to the reinforced stimulus: CS^+^/(CS^+^ + CS˗). A score of 1 reflects perfect discrimination between stimuli, whereas a score of 0.5 reflects no discrimination between CS+ and both CS˗ stimuli.

During the expectancy phase we measured the outcome expectancy to each stimulus (ratings from 0 to 100). This is the critical measure to evaluate the generalization response to all the stimuli tested. This measure was analyzed with two different approaches.

First, during the expectancy test, we used a custom analytical technique designed by Lee et al. (2021) to directly measure the breadth of generalization. This technique involves fitting an augmented Gaussian function to individual generalization gradients (Lee et al., 2021). The augmented function allows for the estimation of various descriptive features of the gradients. We were particularly interested in the breadth of the generalization gradients (i.e., the standard deviation of a Gaussian function, Width Green and Width Blue). Because the augmented Gaussian is the combination of two half-Gaussian functions, Width Green refers to generalization to the stimuli trained on the left side of the CS+ (i.e., green side) and the Width Blue to generalization the stimuli in the right side (i.e., blue side). Posterior estimates in each group will be indicative of a broader response, that is, a higher outcome expectancy to non-trained stimuli.^2^ Frequentist analyses often rely on interactions with quadratic trends, which do not always indicate that one gradient is broader than the other. In contrast, this technique allows us to directly compare the breadth of the gradient between the two groups.

Differences between groups were evaluated for each Width parameter, simulating the group difference by sampling from the group posterior. The group differences in the posterior distribution were captured by the p(direction) statistic. The p(direction) is the proportion of posterior that lies above or below 0, that is, a change in a particular direction. In a situation in which both groups performed similarly and there is a perfect overlap between the posterior distributions, the posterior differences between groups should be centered around 0, and therefore the p(direction) is around 0.5. This indicates that there is a 50% of possibility for the difference between groups being positive or negative. However, if the posterior distribution of groups differed, this would be reflected by values of p(direction) closer to 1, suggesting a group difference with change in the posterior distribution in a particular direction. So far, we expected that Group Trace would exhibit broader gradients, and then we expected to observe a higher p(direction) value, suggesting a change in the posterior distributions between groups. Interestingly, this p(direction) value can be converted into p-value, simplifying the answer of differences between groups. Notably, the interpretation of p(direction) follows a continuous rationale, where values closer to 1 indicate a higher likelihood of a shift in the posterior distribution. To facilitate interpretation, we adopted a threshold of p(direction) > 0.975 as indicative of a significant difference in a two-tailed comparison (Lee et al., 2021; Makowski et al., 2019). This threshold allowed us to identify meaningful differences in breadth between the two groups. However, it is important to interpret this dichotomous decision with caution, as it inherently supports a continuous perspective, contrasting with the dichotomous decision-making framework of frequentist analyses.

The hierarchical model developed by Lee and colleagues (2021) estimates parameters at multiple levels – for subject and group – influencing estimation of one level over the other. In our case, given the use of a double negative discrimination, we fixed the estimation of the peak location of both levels (group and subject) to the CS+. This implies that the peak of the gradients was adjusted to the expected value of the CS+, in the middle of the continuum, and the peak location was not estimated by the model.

Second, we also provided inferential analysis describing the linear and quadratic tendencies in the gradients and the potential modulation by group factor. These additional analyses were included to complement the analyses reported by the augmented Gaussian function (see Lee et al., 2021). The rejection criterion was set at .05 for all statistical tests. Effect sizes and their confidence intervals are reported for tests relevant to the study hypothesis. Confidence intervals on partial-eta squares (95%) were computed using software available in Nelson (2016).

In the identification phase, the proportion of responses to each test stimulus was analyzed exploring differences between groups using a mixed ANOVA with Stimuli manipulated within subjects and the factor Groups as the between-subjects variable.

### Results

#### Training phase

**Fig. 2:**
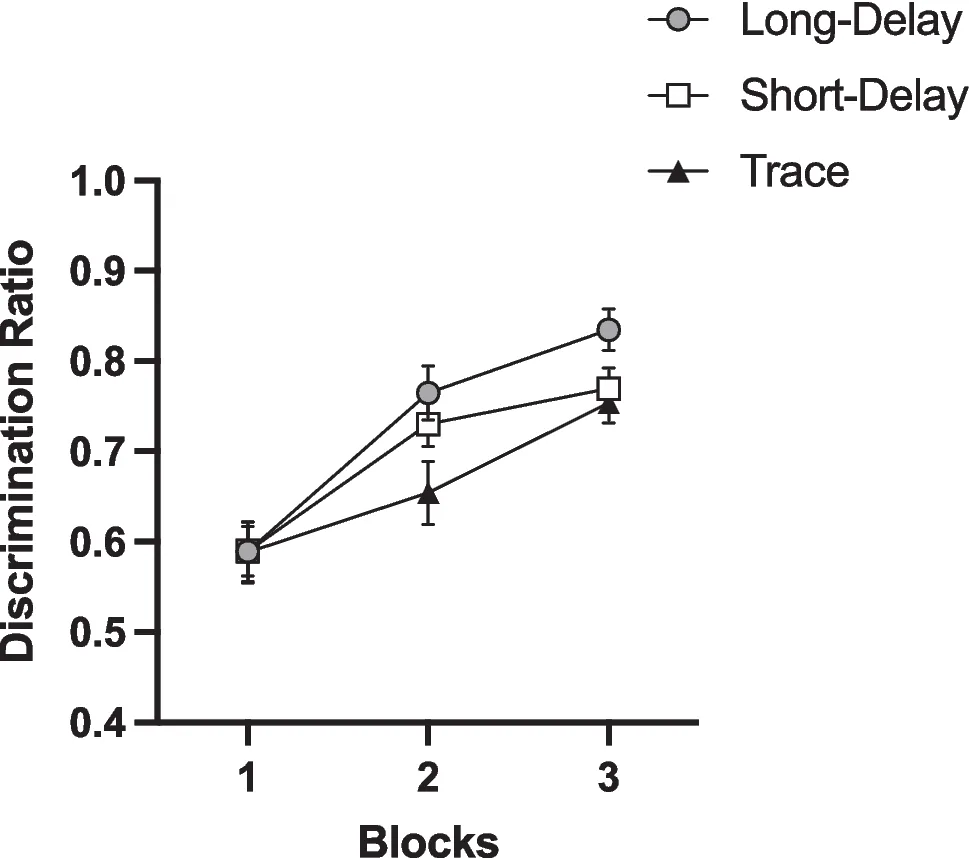
Discrimination ratio in Experiment 1. Error bars represent the SEM

Figure 2 depicts the discrimination ratios across the three blocks of training. In general, all groups increased their discrimination over blocks. A 3 (Group: Long-Delay, Short-Delay, Trace) × 3 Block (1, 2, 3) mixed-ANOVA revealed a main effect of Block, *F*(2, 342) = 68.54, *p* <.001, η^*2*^_*p*_ =.29, 95% CI [.21,.36] but no effect of Group, *F*(2, 171) = 1.92, *p* =.149, nor an interaction, *F*(4, 342) = 2.16, *p* =.073. However, as Fig. 2 suggests, there seem to be differences between groups in the last block of training. Indeed, in the third block, the effect of Group was significant, *F*(2, 171) = 3.50, *p* =.032, η^*2*^_*p*_ =.04, 95% CI [.001,.04]. Tukey post hoc tests revealed differences between Group Trace and Group Long-Delay, *p* =.039, but not between Trace and Short-Delay, *p* =.870. There were no differences between Long-Delay and Short-Delay, *p* =.106.

#### Expectancy test

**Fig. 3:**
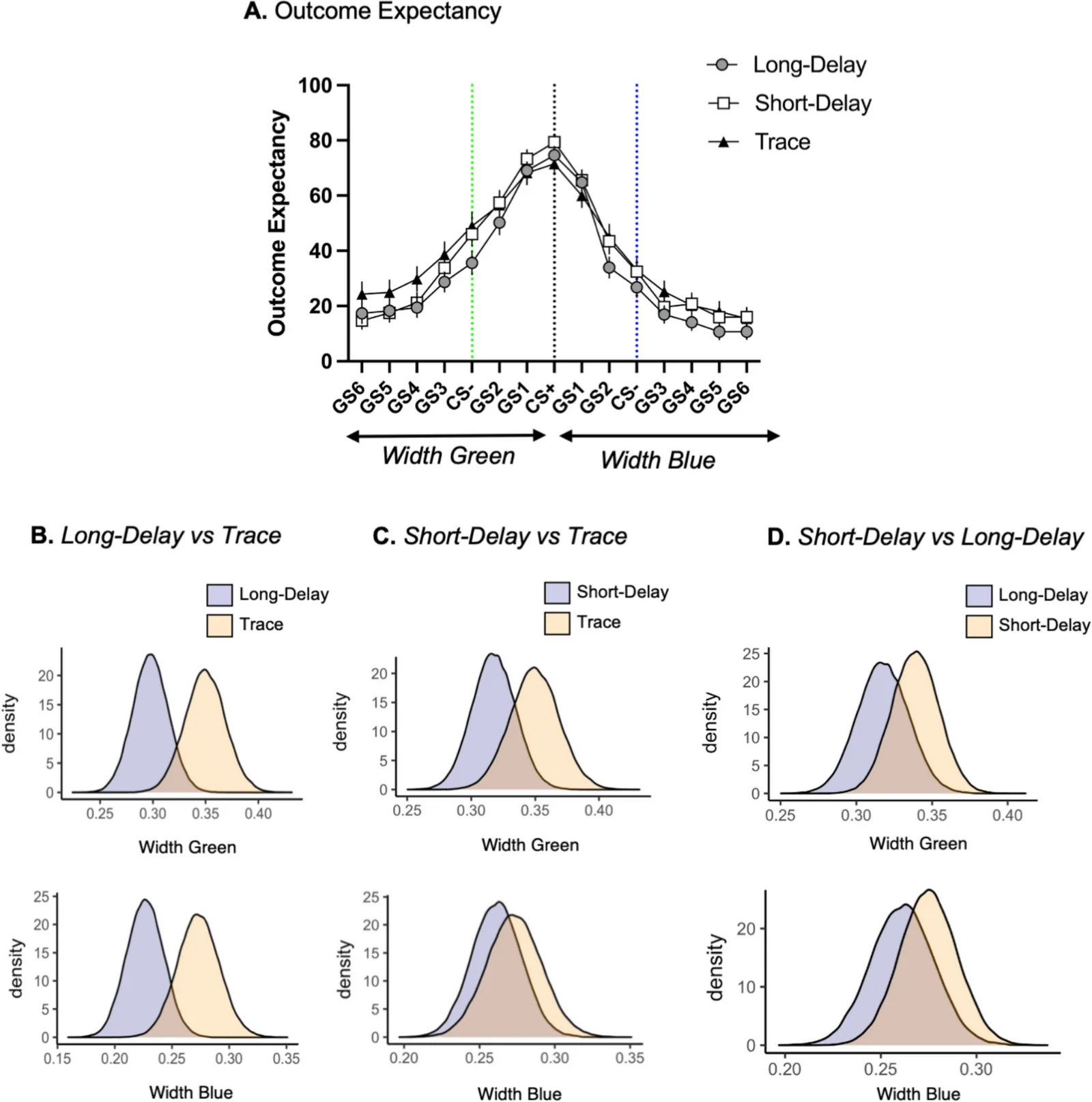
Generalization gradients and density plots in Experiment 1. (**A**) The generalization gradients for all groups. The CS+(S11) was in the middle of the continuum (black dashed line). There was a greener CS˗ (S8) on the left side and a bluer CS˗ (S14) on the right (green and blue dashed lines). GS refers to the Generalization Stimuli. Numbers indicate the proximity to the CS+, GS1 was the closer to the CS+ (S10 and S12) and GS6 was further away (S1 and S21). Note that the left part of the figure shows GS greener than the CS+ and the right side shows GS bluer than the CS+. Error bars represent the SEM. (**B**) Comparison in the Width Green and Width Blue posterior estimates of the augmented Gaussian function of groups Long-Delay and Trace. (**C**) Comparison in the Width Green and Width Blue posterior estimates of the augmented Gaussian function of groups Short-Delay and Trace. (**D**) Comparison in the Width Green and Width Blue posterior estimates of the augmented Gaussian function of groups Long-Delay and Short-Delay

Figure 3A represents the gradient in the three experimental groups. In all groups, outcome expectancy peaked in the CS+ and progressively decreased in both slopes. However, there were slight differences between groups. We analyzed these with the augmented Gaussian function by pairs of groups.

### Augmented Gaussian function

*Long-Delay versus Trace:* Group Long-Delay showed a sharper decrease, with lower ratings in the non-reinforced and all the non-trained stimuli (except those immediately adjacent to the CS+). Figure 3B depicts the breadth of the gradients for the Width Green (upper figure) and Width Blue (lower figure) dimensions using the augmented Gaussian function. In both cases, it is possible to observe higher posterior estimates for group Trace (orange) compared to Long-Delay (blue). Indeed, the *p*(direction) for the Width Green parameter indicated a meaningful change in the direction of the distribution (0.98) above the threshold (0.975); however, this value was not reached (although very close) in the Width Blue (0.97).

*Short-Delay versus Trace:* We compared groups Short-Delay and Trace, and the differences between groups are in the same direction. As Fig. 3C shows, there were slightly broader gradients in Group Trace compared with Group Short-Delay in the Width Green. However, the p(direction) = 0.90 did not reach the threshold. This was also the case in the Width Blue, p(direction) = 0.68.

*Short-Delay versus Long-Delay:* Finally, Fig. 3D represents both Delay groups. As described in Table 1, the comparison between the two groups revealed no differences between them, since in both cases the p(direction) clearly fell below the threshold.

**Table 1:** Summary of posterior odds and p(direction) in specific group comparisons

Experiment	Groups	Parameter	HDI Low	HDI High	p(direction)
1	Long-Delay vs. Trace	Width Green	−0.1	0.001	**0.98**
		Width Blue	−0.09	0.001	0.97
	Short-Delay vs. Trace	Width Green	−0.08	0.02	0.90
		Width Blue	−0.06	0.04	0.68
	Short-Delay vs. Long-Delay	Width Green	−0.06	0.02	0.82
		Width Blue	−0.06	0.001	0.73
2	Long-Delay vs. Trace	Width Green	−0.14	−0.05	**1.00**
		Width Blue	−0.07	0.02	0.87
	Short-Delay vs. Trace	Width Green	−0.11	−0.01	**0.99**
		Width Blue	−0.04	0.05	0.62
	Short-Delay vs. Long-Delay	Width Green	−0.08	0.01	0.94
		Width Blue	−0.08	0.01	0.93

The threshold to consider that p(direction) was different between groups was 0.975. Bold values represent that the difference reached the threshold. Width Green refers to the side in which the CS˗ was greener than the CS+, and Width Blue to the side in which the CS˗ was bluer than the CS+

#### Frequentist analyses

A 3 (Group: Long-Delay, Short-Delay, Trace) × 15 Stimuli showed a quadratic component, *F*(2, 171) = 455.41, *p* <.001, η^*2*^_*p*_ =.84, 95% CI [.80,.87], that did not interact with Group, *F*(2, 171) = 1.23, *p* =.294. Moreover, the effect of group was not significant, *F*(2, 171) = 2.73, *p* =.068. Similar to the previous analyses, we analyzed each side of the continuum. Analyses of the Width Green did not reveal a main effect of Group nor Group × Stimuli interaction, with the largest *F* for the main effect of Group, *F*(2, 171) = 1.66, *p* =.193. Analyses on the Width Blue showed a similar pattern. There was no main effect of Group nor Group × Stimuli interaction, largest F for the main effect of Group, *F*(2, 171) = 1.38, *p* =.253.

#### Identification test

**Fig. 4:**
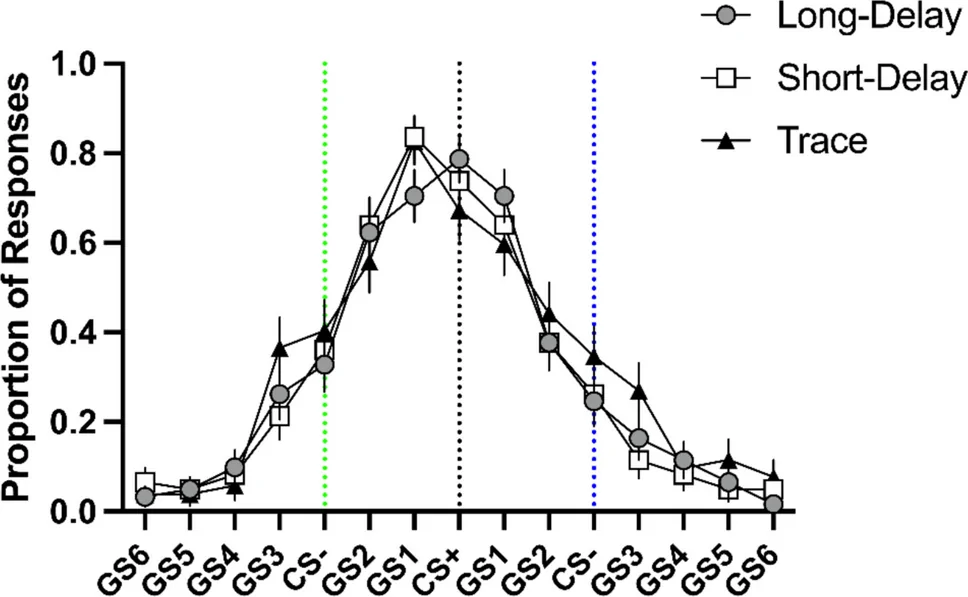
Identification test in Experiment 1. The CS+ (S11) was in the middle of the continuum (black dashed line). There was a greener CS˗ (S8) on the left side and a bluer CS˗ (S14) on the right (green and blue dashed lines). Error bars represent the SEM

Figure 4 depicts the identification response of participants to the 15 stimuli. A 3 (Group: Long-Delay, Short-Delay, Trace) × 15 Stimuli revealed an effect of Stimuli, *F*(14, 2394) = 91.83, *p* <.001, η^*2*^_*p*_ =.35, 95% CI [.32,.37]. The Group × Stimuli interaction was not significant, *Fs* < 1. Overall, the proportion of same responding was higher in the green side compared to the blue side *F*(1, 173) = 6.81, *p* =.010, η^*2*^_*p*_ =.04, 95% CI [.001,.11] suggesting a bias for misidentifying the stimuli on the green side.

### Experiment 1: Discussion

Experiment 1 provided partial support for the idea that weakening temporal contiguity broadens generalization gradients. This overgeneralization by group Trace on outcome expectancy was evident in the comparison with group Long-Delay in the green side of the gradient, and slightly weaker in the blue side. In the case of group Short-Delay, this pattern was not observed. Nevertheless, if anything, this difference was apparent in the green side, but not in the blue side. The differences between green and blue sides may be driven by a poorer identification of the greener stimuli, as the higher identification response in the greener side during identification test suggests. We discuss this asymmetry further in the *General discussion*.

As posited in the *Introduction*, we included two control groups because each group enabled us to control for different behavioral aspects during learning. In this case, it seems that the differences observed between Groups Trace and Long-Delay might be driven by the advantage derived from longer exposures to the stimuli in Group Long-Delay. Indeed, at the end of training Group Trace had poorer discrimination compared to Group Long-Delay. However, as was revealed by the identification test, both groups showed similar performance, suggesting that differences during outcome expectancy at test were not merely driven by differences in identification of the stimuli.

Looking at the results revealed by the augmented Gaussian function, group Trace showed a tendency towards broader generalization compared to both Delay groups (see density plots for each width of the gradient). However, only in the case of the green side and compared to Long-Delay did this difference reach the threshold considered as significant. One potential reason for the low sensitivity might be the type of dependent variable used during expectancy test phase. The change in the structure of the task and in the dependent variable from training to test may have decreased sensitivity to observe differences between groups. Experiment 2 was run to replicate the results and test this possibility.

## Experiment 2

In the generalization test of Experiment 1, outcome expectancy was recorded using a rating scale. However, during training, it was the rate of pressing that was used as an index of outcome expectation. It could be the case that this change from training to test decreased sensitivity to observe differences between groups, because the number of presses might not directly correlate with a given value in the expectancy ratings. This change should make the expectancy test more similar to training, avoiding a potential generalization decrement by changes in the structure of the task.

Hence, Experiment 2 replicated Experiment 1, but generalization during the tests was assessed using the same response as training. Extending the previous finding to a new measure will also test the robustness of the putative effect.

### Participants

Two hundred and fifty participants (126 female, 121 men, two non-binary, one prefer not say) with an average age of 37.04 (*SD* = 12.38, range 19–72) years were recruited via Prolific in exchange for £2.9. Participants had no previous experience with the task. They were required to have completed more than 200 studies in Prolific with an approval rate > 98% and to be fluent in English language.

### Procedure

The experiment was identical to the previous one, except for the way of assessing generalization during the expectancy test. In the new Expectancy test, participants continued visualizing the stimuli on the machine and pressing as they did during training. Hence, the stimulus appeared on the screen for the same amount of time as during training (4 s for group Short-Delay and Trace and 12 s for Long-Delay). Because participants were instructed that there was no outcome during the test phase, the same ITI for all groups (8 s) was used in order to avoid unnecessarily extending the length of the test phase. The instructions for the expectancy phase were modified slightly relative to the previous experiment:

*For the next phase of the experiment, you will be shown some more signals, and you should continue pressing the button to alert the security team about whether you think lightning will occur. You should make your predictions based on what you have learned during the previous phase. However, in this phase, you will NOT see any lightning occurring. Please respond as if the lightning could occur.*

*Once again, the number of presses should reflect the strength of your belief about whether lightning is going to occur: the more you press the button, the more you believe lightning is coming.*

### Data analysis

During the expectancy phase we recorded the number of presses during each stimulus presentation. Because different groups had a different stimulus duration, we used relative responses to compare the generalization gradients. The relative responses were calculated by dividing the number of responses in each stimulus by the maximum number of responses in a given stimulus during test,^3^ This way, the stimulus recruiting the largest number of responses had a value of 1 and the rest of the stimuli were in proportion to that stimulus. Finally, each value was multiplied by 100 to make the gradients comparable to the previous experiment (see Mackintosh, 1974).

### Results

#### Training phase

**Fig. 5:**
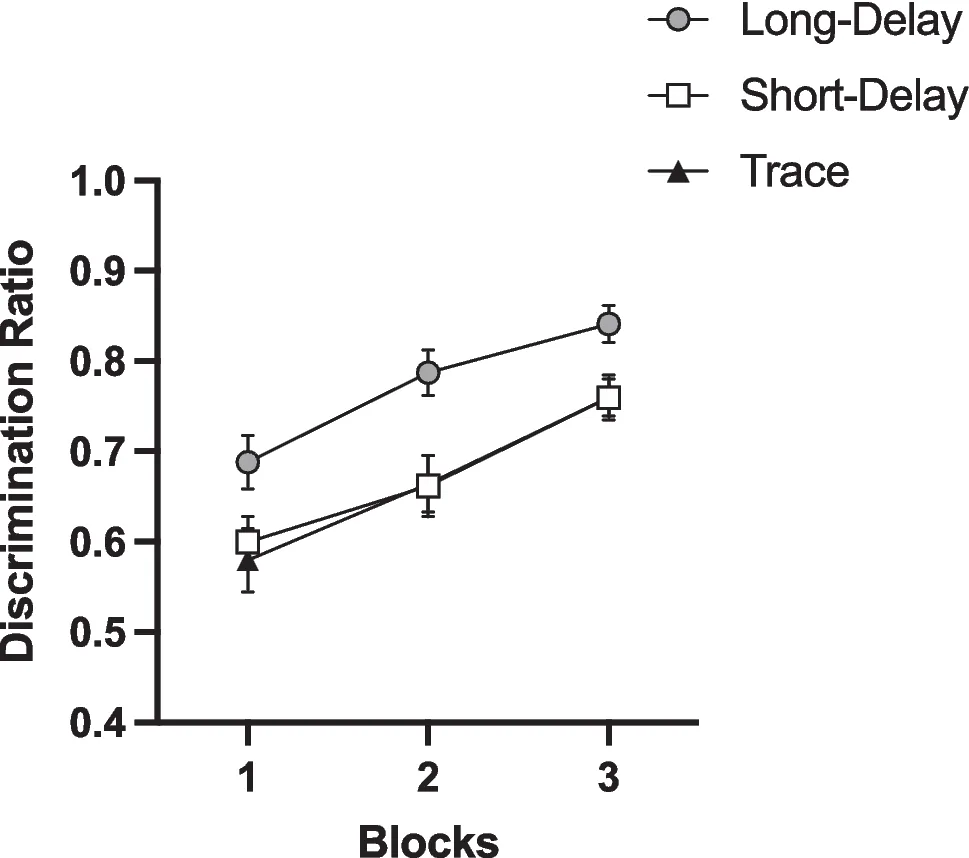
Discrimination ratio in Experiment 2

Figure 5 depicts the discrimination ratio across the three blocks of training. In general, the discrimination scores increased in all groups over blocks; however, it is clear that Group Long-Delay performed better than the other two groups. A 3 (Group: Long-Delay, Short-Delay, Trace) × 3 Block mixed-ANOVA revealed a main effect of Block, *F*(2, 360) = 47.13, *p* <.001, η^*2*^_*p*_ =.21, 95% CI [.14,.27] and a main effect of Group, *F*(2, 180) = 7.80, *p* =.001, η^*2*^_*p*_ =.08, 95% CI [.02,.16] but no Group × Block interaction, *F*(4, 360) = 0.64, *p* =.633. Tukey post hoc tests during the last block of training revealed differences between group Long-Delay and group Trace, *p* =.001, and groups Long-Delay and Short-Delay, *p* =.005, but not between groups Trace and Short-Delay, *p* =.918. Overall, group Long-Delay performed better than the other two groups.

#### Expectancy phase

**Fig. 6:**
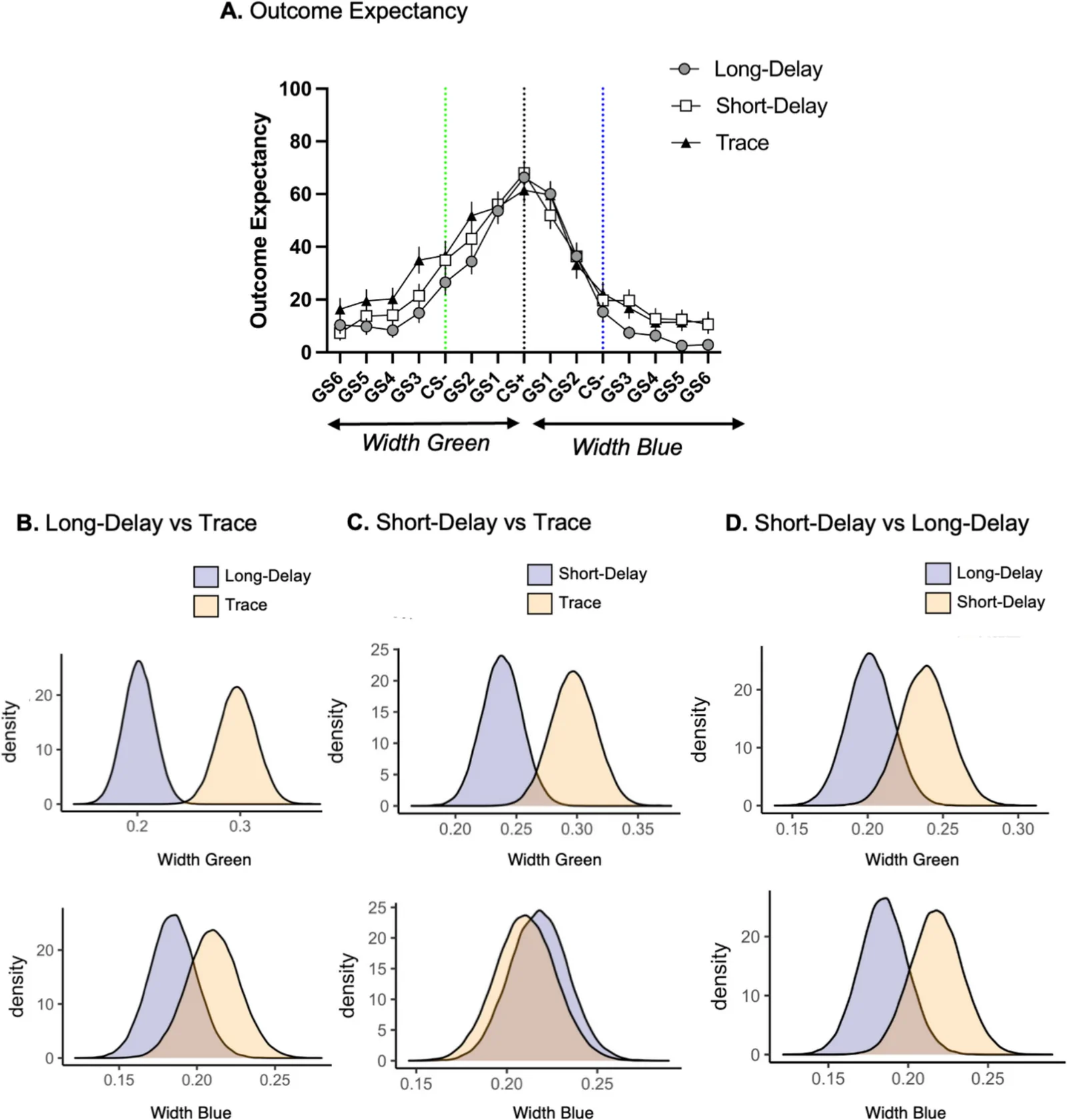
Generalization gradients and density plots in Experiment 2. (**A**) The generalization gradients for all groups. The CS+ (S11) was in the middle of the continuum (black dashed line). There was a greener CS˗ (S8) on the left side and a bluer CS˗ (S14) on the right (green and blue dashed lines). GS refers to the Generalization Stimuli. Numbers indicate the proximity to the CS+, GS1 was the closer to the CS+ (S10 and S12) and GS6 was further away (S1 and S21). Note that the left part of the figure shows GS greener than the CS+ and the right side shows GS bluer than the CS+. (**B**) Comparison in the Width Green and Width Blue posterior estimates of the augmented Gaussian function of groups Long-Delay and Trace. (**C**) Comparison in the Width Green and Width Blue posterior estimates of the augmented Gaussian function of groups Short-Delay and Trace. (**D**) Comparison in the Width Green and Width Blue posterior estimates of the augmented Gaussian function of groups Long-Delay and Short-Delay

Similar to the previous experiment, Fig. 6A shows that the peak of the response was in the CS+ in all groups and progressively declined to both sides.

#### Augmented Gaussian function

*Long-Delay versus Trace:* Figure 6A suggests a broader generalization in group Trace compared to group Long-Delay. Differences between groups were evident in the Width Green, in which, as Fig. 6B shows, there was no overlap between the two distributions, *p*(direction) = 1. Indeed, the *p*(direction) is above the threshold supporting significant differences between the two groups. However, on the other side there was more overlap, *p*(direction) = 0.87, indicating no significant differences. In general, the pattern of results was very similar to the first experiment, with significant differences in the green side (Width Green), but not in the blue side (Width Blue).

*Short-Delay versus Trace:* In the case of groups Trace versus Short-Delay, Fig. 6C indicates a somewhat similar pattern. As we can see in the upper panel, there was a broader response in group Trace, but only in the Width Green (green side), with a *p*(direction) = 0.99 above the threshold. However, in the Width Blue there were no differences between the two groups, *p*(direction) = 0.62.

*Short-Delay versus Long-Delay:* We did not find differences in the case of Groups Short-Delay versus Long-Delay (Fig. 6D), either in the Width Green *p*(direction) = 0.94 or in the Width Blue, *p*(direction) = 0.93. If anything, group Short-Delay tended to show a broader gradient in both sides.

In this experiment, group Long-Delay showed better discrimination than the other two groups at the end of training. However, during the generalization test, group Short-Delay was not different from group Long-Delay, but group Trace was. Therefore, the differences at the end of training were insufficient to explain the pattern observed during the generalization test, because groups Trace and Short-Delay discriminated similarly at the end of training, but they showed some differences during the generalization test.

#### Frequentist analyses

A 3 (Group: Long-Delay, Short-Delay, Trace) × 15 Stimuli revealed a quadratic component for the main effect of Stimuli, *F*(1, 180) = 373.25, *p* <.001, η^*2*^_*p*_ =.67, 95% CI [.60,.73] but not the interaction by Group, *F*(2, 180) = 0.56, *p* =.571. The main effect of Group was significant, *F*(2, 180) = 5.33, *p* =.0061, η^*2*^_*p*_ =.05, 95% CI [.01,.13]. Similar to the first experiment and for consistency with the augmented Gaussian function, we analyzed the effect of group in each side. Analyses of the Width Green revealed a main effect of Group, *F*(2, 180) = 4.64, *p* =.011, η^*2*^_*p*_ =.05, 95% CI [.001,.12]. Tukey post hoc tests indicated differences between Groups Trace and Long-Delay, *p* =.008, but not with Short-Delay, *p* =.201 groups. The effect of Group was not significant in the Width Blue, *F*(2, 180) = 1.73, *p* =.179. Tukey post hoc tests did not reveal differences between Groups Trace and Long-Delay, *p* =.222, nor with Short-Delay, *p* =.989.

In general, the augmented Gaussian function seemed more sensitive to capturing the differences between groups. For example, detecting differences between Trace and Short-Delay in the Width Green, something that was not supported by frequentist analyses.

#### Identification phase

**Fig. 7:**
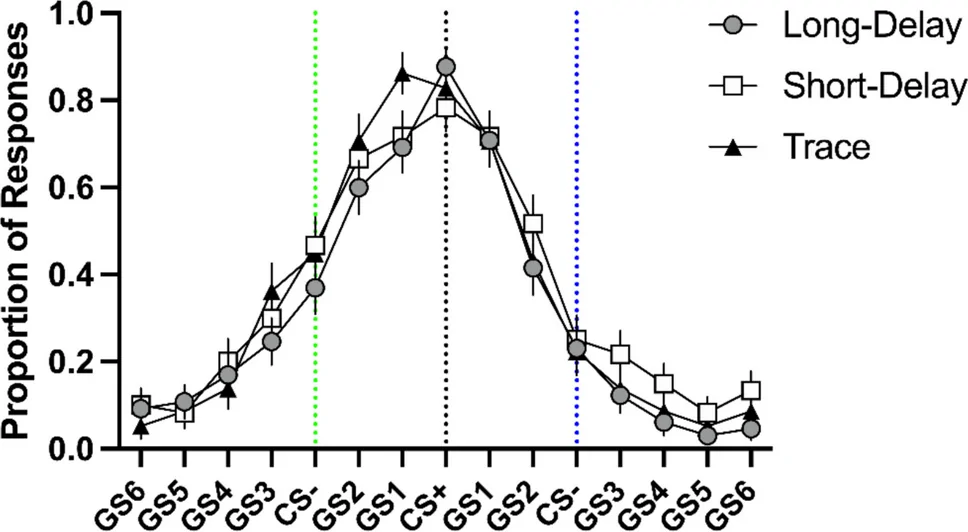
Identification in Experiment 2. The CS+ (S11) was in the middle of the continuum (black dashed line). There was a greener CS˗ (S8) on the left side and a bluer CS˗ (S14) on the right (green and blue dashed line). GS refers to the Generalization Stimuli. Numbers indicate the proximity to the CS+, GS1 was closer to the CS+ (S10 and S12) and GS6 further away (S1 and S21). Note that the left part of the figure shows GS greener than the CS+ and the right side shows GS bluer than the CS+. Error bars represent the SEM

A 3 (Group: Long-Delay, Short-Delay, Trace) × 15 Stimuli only revealed a main effect of Stimuli *F*(14, 2520) = 103.90, *p* <.001, η^*2*^_*p*_ =.36, 95% CI [.34,.39] but neither the main effect of Group nor the interaction were significant, largest *F*(2, 180) = 1.01, *p* =.368 for the main effect of Group. Overall, the rate of responding was higher in the left side compared to the right side, *F*(1, 180) = 14.38, *p* <.001, η^*2*^_*p*_ =.07, 95% CI [.02,.16] suggesting again a bias for identifying the stimuli on the green side (see Fig. 7).

## StephensGeneral discussion

Across two experiments, we evaluated whether weakening of temporal contiguity broadens generalization gradients in human predictive learning. In general, the shape of gradients during test was consistent with the double negative discrimination used during training, with outcome expectancy peaking at the CS+ and progressively decreasing as a function of perceptual similarity of the non-trained stimuli on both sides of the gradient. In line with the initial predictions, there was a tendency for broader gradients in group Trace compared to group Long Delay (Experiment 1 and 2) and group Short-Delay (only in Experiment 2). Importantly, this pattern was found using two different ways of assessing outcome expectancy. Nevertheless, this pattern was reliable in the green side of the continuum, leaving the blue side relatively unaffected by weakening of temporal contiguity.

An apparent reason for the absence of a more robust effect of trace may be due to the relative short trace (8 s). It is well known that behavioral control weakens as trace length increases. For example, in animal research, it is common to use traces of 30 s or more (e.g., Honey & Hall, 1992; Marlin, 1981; Williams, 1999). Hence, longer traces might have had a larger impact on behavioral control by the stimuli. However, because the experiments were conducted online, we did not want to use very long traces, in order to avoid shifts of attention during the trace interval. It is possible that in a more traditional laboratory setting, participants will be more committed to the experimental manipulation and better engage with the task when using longer traces. In any case, the length of trace used here has proved to be effective in other learning paradigms (Herrera et al., 2022; Shanks & Dickinson, 1991; Maddox & Ing, 2005), but it might be necessary to use a longer trace to get a stronger effect on generalization gradients.

Additionally, in a double-negative discrimination such as the one used here, gradients obtained at test may reflect both excitation from the CS+ and or inhibition from the CS– stimuli. Both CS˗ stimuli likely generated inhibitory gradients that could summate with the excitatory gradient (Spence, 1937), yielding a net response determined by the balance between these opposing excitatory and inhibitory tendencies. This might complicate interpretation of the data, as broader responding could, in principle, result either from a broader excitatory gradient around the CS+ and/or from a weaker inhibitory generalization around the CS˗. One could argue that the observed broadening of the excitatory gradient in trace conditioning is a by-product of a narrower inhibitory gradient, leading to reduced suppression and thus an apparent enhancement of excitation. However, existing evidence indicates that inhibition typically generalizes more broadly than excitation (Rescorla, 2006; see also Urcuioli & Honigi, 1980). Consequently, it seems unlikely that the broader gradients observed under weak temporal contiguity merely reflect reduced inhibitory generalization. If anything, broader inhibitory generalization would be expected to counteract, rather than strengthen, the excitatory broadening observed in our experiments.

Furthermore, following the logic of our theoretical rationale, a trace manipulation could plausibly induce even a broader inhibitory gradient leading to greater overlap and interference between excitatory and inhibitory gradients. Yet this account also appears difficult to reconcile with the current pattern of results, as the broader excitatory gradient was observed with weak temporal contiguity. Having said that, with the present design it is not possible to completely rule out a contribution of inhibition by the CS˗ stimuli, although it should be mentioned that parallel work in nonhuman animals has revealed broader gradients with trace conditioning in the absence of CS˗ stimuli presented during training (Marchand & Camper, 2000). Nevertheless, this possibility highlights an interesting direction for future research. To the best of our knowledge, no study has directly examined how temporal contiguity modulates the generalization of inhibition, and such work could shed light on the interplay between excitatory and inhibitory processes.

In these experiments, because we used a double-negative discrimination design, it is important to note that a classic peak-shift effect was not expected. Peak shift typically arises when excitation from a single CS+ interacts asymmetrically with inhibition from a single CS˗ positioned on one side of the same dimension, resulting in a change in the peak of responding away from the CS˗ (Akins et al., 1981; Gallaghar et al., 2020; Hanson, 1959). According to standard accounts of generalization and the peak shift (Spence, 1937), when two inhibitory stimuli symmetrically flank the CS+, as in the present experiments, the resulting inhibitory gradients should cancel each other, resulting in no peak shift. Consequently, the net function should remain centered on the CS+, producing a symmetric pattern rather than a directional shift. Similarly, according to rule-based accounts of the peak-shift phenomenon, when training of a CS+ occurs along with two CS˗ stimuli, the evidence does not support inference of a relational rule, which is critical – in combination with participants who adopt a similarity rule – to observe the peak shift in humans (Lee et al., 2018). Supporting this interpretation, Ng et al. (2022) found no evidence of peak shift in a human double-negative design, further suggesting that symmetry in inhibitory placement minimizes displacement effects. The absence of a peak-shift effect may provide additional support for the idea that the two inhibitory gradients counteracted one another, and consequently, the resulting gradient is more likely to reflect the profile of the excitatory gradient alone (see above). In any case, future studies may want to disentangle the specific contribution of excitatory and inhibitory gradients in the broader generalization observed in our experimental design.

Across both experiments, we observed an asymmetric pattern in the gradient, with more generalization to and poorer identification of stimuli greener than the aqua CS+. It could be the case that discrimination for the green stimuli was more difficult due to some arbitrary perceptual properties. Because the experiments were conducted online, we could not control participants’ display calibration or indeed ambient lighting conditions. Therefore, this variability may have introduced perceptual noise, particularly affecting the fine perception of hue differences in the green side of the blue-green dimension. This limitation should be considered when interpreting the absolute steepness of the gradients; however, any such variability is likely to have been randomly distributed across conditions and therefore balanced across groups. Moreover, numerous online studies using similar color-based continua (e.g., Lee et al., 2018; Lovibond et al., 2020; Zaman et al., 2023) have reported highly systematic generalization gradients that closely align with the experimental manipulations, suggesting that these effects clearly reflect the underlying learned processes rather than minor perceptual variations. Having clarified that, it could be the case that this artifact somehow triggered a more challenging discrimination on the green side of stimuli. This difference was consistently observed, even though the greener and the bluer CS˗ were equally distributed in the hue dimension. Beyond perceptual properties of stimuli, at a speculative level, it could be the case that given that the task was embedded in a continuous pressing task, the greener stimuli were inherently associated with a “go” response, increasing the rate of pressing for green stimuli during test. In the Online Supplementary Materials, we provide the rate of responses during training distributed per second of each CS. It is interesting to note that the response to the green CS˗ was higher in all groups compared to the blue CS˗. This difference is consistent with the idea that it was easier to respond to a green cue.

In any case, the asymmetry between both slopes should not undermine the fact that gradients resulted in the expected bell shape, it just seems that identification of stimuli on the green side may be more difficult, as seen by the asymmetry in the identification phase in both experiments. Indeed, during the generalization phase, we observed that group Trace showed broader gradients compared to groups Short-Delay and Long-Delay in the green side, but not in the blue side. However, during the identification test we did not find differences between groups, suggesting that the level of identification of the target CS+ was similar across groups. This pattern supports the idea that the higher outcome expectancy was not merely driven by loss of perceptual discrimination (Zaman et al., 2021), and hence that other factors could be affecting the broader gradients observed in Group Trace (e.g., less confidence about their judgments, see Online Supplementary Material).

Indeed, in the case of Short-Delay versus Trace, both groups performed similarly at the end of training and in the identification phase, but not in the expectancy phase (at least in the green side in Experiment 2). Therefore, the broader gradient observed in the green side is unlikely to have resulted exclusively from poor discrimination during training or identification during test. Consistent with this, a recent study by Ram et al. (2024) found that including a fictitious temporal gap also yielded broader response. In a predictive learning scenario, participants initially learned a specific set of associations, and afterwards, they were required to apply this learning either to an immediate situation or to one occurring in the more distant future. They found a wider response in the situation in which they increased psychological distance (distant future). In other words, anticipating a longer temporal gap between learning and its future application increased the breadth of generalization. This pattern is particularly interesting because participants in both conditions demonstrated the exact level of discrimination at the end of training and were tested at the same time (immediately after training). However, the mere expectation of an immediate versus a delayed application was sufficient to broaden the generalization gradient. Taken together, temporal expectations impact generalization beyond perceptual properties, highlighting the role of time in shaping learned responses.

Overall, we found a very similar pattern of results across both experiments. However, in Experiment 1 there were two potential sources of generalization decrement from training to test. First, during the generalization test we assessed outcome expectancy using a rating scale rather than an instrumental response. Second, there was a change in stimulus duration from training to test, because during training the exposure was fixed, but during testing each participant was exposed to the stimulus for the time required to respond. In Experiment 2, we attempted to control these two sources of generalization decrement by using the same instrumental response during both training and testing, and by keeping the duration of the stimuli during testing identical to that used in training. This change reduced potential confounds arising from these sources of generalization decrement as occurred in the first experiment. As a counterpart, however, the stimuli had different durations, potentially introducing confounds during testing. This is particularly relevant in the case of trace conditioning and stimuli with long exposure, in which the temporal learning is a key factor (Gallistel & Balsam, 2014), Nonetheless, we believe this drawback is less problematic than the alternative of changing stimulus duration between phases, and applies to all groups. The use of relative gradients helped to normalize the overall pattern of responses. In any case, future studies may need to examine more deeply how temporal features of learning interact with generalization processes.

Historically, two theoretical frameworks have been proposed to account for the effect of trace conditioning (Boakes & Costa, 2014). On the one hand, “decay theories” assume that stimuli features that are present at the time of termination of the CS+ presentation progressively fade out after offset of the stimuli, and consequently, when the outcome is presented, the number of stimulus elements which are active is lower in trace than in delay conditioning procedures, and this accounts for the observed behavioral differences (e.g., Rawlins, 1985). Indeed, the absence of recent perceptual features may induce participants to misperceive the CS+. Hence, according to a pure decay explanation, poorer performance would be expected in group Trace in the identification test. However, in all experiments we observed similar identification accuracy regardless of temporal contiguity, suggesting that the different expectancy gradients were not merely driven by perceptual properties and other factors may contribute to the broader generalization. One possibility is concerned with the perceived level of temporal uncertainty. In this experimental series, the trace used was fixed; however, future studies may want to study the effect of trace including additional factors, such as temporal predictability (Greville & Buehner, 2010), instructions provided (Buehner & May, 2004), or the presence of intervening events (Costa & Boakes, 2011; Lagnado & Speekenbrink, 2010). All these factors increased the deleterious effect of weakening temporal contiguity, and might provide a better scenario to observe a more consistent effect.

A second theoretical framework that has been proposed to account for the effect of trace intervals is the interference framework. According to this principle, intervening events during trace interval interfere with the target cue as potential predictors of the outcome (Revusky, 1971). Despite the fact that we used a “clean” trace without any intervening events, participants were allowed to make responses during the trace, resulting in some degree of interference. There was a response-interference context, since the motor response might interfere with maintaining the representation of stimuli during the trace (Lieberman et al., 2008). Similarly, with manipulations directly related to the length of trace and decay information, future studies may extend the implications of trace conditioning in determining the breadth of the gradient in the presence of intervening events (e.g., Costa & Boakes, 2011; Lagnado & Speekenbrink, 2010), or with the use of a secondary task varying the cognitive demands (e.g., Carter et al., 2003).

Admittedly, the current experimental series was not aimed at distinguishing between these general frameworks; instead these studies were designed to assess in human participants whether weakening temporal contiguity results in a broader response in the presence of novel stimuli. As mentioned in the *Introduction*, there are instances in which variations of temporal contiguity have resulted in counterintuitive effects on behavioral control, for example, in the context of cue interaction phenomena (Alcalá et al., 2023; Herrera et al., 2022; Urcelay, 2017; Urcelay & Miller, 2009). Considering cue interaction as an instance of generalization decrement (Pearce, 1987), we hypothesized that the shift from overshadowing to potentiation may be related to the degree of generalization occurring (see Herrera et al., 2022). In the present study, under certain conditions, the generalization response was broader with trace conditioning, indicating a smaller generalization decrement from the trained stimulus (CS+) to novel stimuli. This effect can be linked to observed cue-interaction phenomena, where the generalization decrement decreased as the temporal contiguity between cues and the outcome weakened.

Overall, the extent to which prior knowledge generalizes to new stimuli has implications in many areas of our life. For example, overgeneralization has been suggested as one critical factor in the study of anxiety. That is, studies have documented overgeneralization in anxiety, and recent meta-analyses show a reliable weak to medium effect size in healthy participants but who are high in trait anxiety (Sep et al., 2019), and indeed in participants with a clinical diagnosis of anxiety (Cooper et al., 2022; Fraunfelter et al., 2022; Lissek et al., 2014). Thus, generalization can be adaptative or maladaptive depending on individual differences and circumstances of daily life. Given the relevance of generalization, many efforts have been deployed to characterize factors that determine generalization, as this may help to understand how it can become maladaptive (see Beckers et al., 2023). However, most studies exploring generalization gradients in animals (see Mackintosh, 1974) and humans (Dunsmoor et al., 2017; Dunsmoor & LaBar, 2013; Lee et al., 2018; Livesey & McLaren, 2009; Lovibond et al., 2020; Meulders et al., 2013; Ram et al., 2019; Wang et al., 2021; Wong & Beckers, 2021; Wong & Lovibond, 2018) use strong temporal contiguity between events and therefore do not address what happens in cases of weak temporal contiguity. This is not trivial, for in many examples of daily life – and indeed in the case of maladaptive responding observed in individuals with anxiety – the dreaded adverse consequences may occur after a certain amount of time has passed rather than immediately. For example, after a dog bite, an individual might experience anxiety symptoms sometime after the incident. In the moment of being bitten, the often observed reaction is to feel fear and try to flee. However, in the experience of anxious symptoms, such as prospective avoidance of the stimuli related to the dog bite, there is a temporal gap between the experience of fear and the potential encounter of the aversive consequence. Hence, a better understanding of the effect of temporal contiguity seems critical in those circumstances. Indeed, given the relationship between anxiety and overgeneralization, the role played by temporal contiguity seems a promising avenue to help to further understand this interplay. Overall, the current study paves the way for a better understanding of the role of contiguity in generalization.

## Data Availability

The data reported in this paper are available at: https://doi.org/10.17639/nott.7575.

## Footnotes

1 This instruction aims to provide the button with properties related to the temporal and likelihood appearance of the outcome in real time, mimicking to some extent the lever that a rat presses in a Skinner box.

2 Despite the fact that the augmented Gaussian function also provided posterior estimates for other parameters such as the Mean (useful to detect peak shift), we did not include these on the reported analyses for simplicity. For example, we did not have any expectations about how trace conditioning might modulate the peak shift. Moreover, we intentionally adopted a design that should not result in a peak shift, because the use of two CS˗ stimuli equidistant from the CS+ create two independent inhibitory gradients, which counteract each other, and therefore the excitatory gradient was expected to be symmetric and peak-shift effects should be eliminated (Ng et al., 2022).

3 As expected, the CS+ was the stimulus that received the highest proportion of maximum responses, with 28.5% of participants showing their peak responding to it. Overall, the stimuli located between the two CS– stimuli accounted for 73% of all maximum responses.

## Declarations

### Open practices statement

The experiments reported in this article were not formally preregistered.
